# Preparedness of primary health care workers and audit of primary health centres for *newborn* resuscitation in Port Harcourt, Rivers State, Southern Nigeria

**DOI:** 10.11604/pamj.2020.36.68.22164

**Published:** 2020-06-04

**Authors:** Datonye Christopher Briggs, Augusta Unoma Eneh

**Affiliations:** 1 Department of Paediatrics, University of Port Harcourt Teaching Hospital, Port Harcourt, Nigeria

**Keywords:** Primary health care workers, primary health centre, neonatal resuscitation, equipment, audit, Rivers state, Nigeria

## Abstract

**Introduction:**

Nigeria still has high newborn deaths and birth asphyxia remains a major cause. Birth attendants´ readiness to perform newborn resuscitation depends largely on their competence in basic resuscitation and availability of newborn resuscitation equipment to enable the various steps outlined in resuscitation guidelines to be applied quickly and appropriately. This study aimed to assess primary health care workers´ experience of neonatal resuscitation and audit primary health centres for availability of neonatal resuscitation equipment.

**Methods:**

this descriptive cross-sectional study surveyed 106 primary health care workers (22 doctors, 84 nurses) randomly selected from 28 Primary Health Centres to document their experiences in newborn resuscitation and appraise the centres for availability of newborn resuscitation equipment. Experience in newborn resuscitation was obtained using a semi-structured questionnaire and audit was with a Proforma following on-site facility visits. Data was analysed using SPSS v20 and displayed in tables and graphs.

**Results:**

all health care workers had resuscitated newborns but only 58(57.4%) had ever used a bag and mask, 53(50%) used stethoscopes and 19(17.9%) had resuscitation protocol in their facilities. Fifteen (53.6%) health centres had functional newborn-specific bag and masks, 11(39.3%) had suction machines and 5(25%) had empty oxygen cylinders.

**Conclusion:**

primary health care workers´ experience of newborn resuscitation is very limited and some primary health centres were grossly unequipped. Neonatal resuscitation training interventions and supplies of neonatal resuscitation equipment are urgently needed.

## Introduction

Globally, birth asphyxia contributes about a third of all newborn deaths with over sixty percent occurring in sub-Saharan Africa where birth attendants skilled in newborn resuscitation are often not present and the necessary tools needed are usually not available [1-3]. In Nigeria birth asphyxia related deaths remains high at 31% [2]. These newborn deaths happen mostly at the community level where unsupervised deliveries occur at home, unorthodox centres with traditional birth attendants or at primary health care centres [4-6]. In these situations, birth attendants with little or no training in basic neonatal resuscitation may perform practices that delay effective ventilation [4-6]. Neonatal resuscitation is a simple intervention shown to significantly reduce newborn asphyxia related deaths by about 30% [7-9]. Nigeria´s determination to reduce its high newborn asphyxia-related deaths adapted Basic/Advanced Neonatal Resuscitation Training (NRT) principally organized by the Paediatric Association of Nigeria (PAN) and the Helping Babies Breathe (HBB), a module in the Essential Newborn Care Course (ENCC) principally organized by the Federal Ministry of Health in collaboration with UNICEF [10]. These courses give health workers the prerequisite training to improve competence in neonatal resuscitation and has been encouraged at all levels of healthcare [10, 11].

Birth asphyxia requires simple and cost effective tools like resuscitator (bag and mask) and suction devices to prevent its occurrence [12]. Nationwide surveys that assessed emergency obstetrics and newborn services in some health facilities in Africa and Asia identified unavailability of equipment, health care workers´ ignorance and unskillfulness as setbacks to perform newborn resuscitation [13-16]. A multi-country survey by Enweronu-Laryea *et al*. [17] involving 12 Low and Middle Income Countries including Nigeria which aimed to grade the “bottlenecks” hindering scale up of basic newborn care and neonatal resuscitation in these countries showed that for neonatal resuscitation, lack of available bag and mask, inadequate workforce and poor service delivery were the major blocks in the health system. In Nigeria, similar shortages of the necessary equipment for neonatal resuscitation have been identified in health facilities [6, 18, 19] but focus on primary health care centres as first level healthcare are scarce. The Primary Health Care Centres (PHC Centres) in Nigeria as conceptualized by the Alma-Ata Declaration of 1978 is considered the model approach to sustainably solve health care challenges. The PHC approach is foundational to achieve universal health coverage and the health-related Sustainable Development Goals (SDGs) [20]. The Rivers State Government had in recent times made tremendous efforts to refurbish previously dilapidated PHC Centres and equipped some model PHC Centres in view of providing extensive healthcare services at the community level [21]. The PHC Centres are run by skilled birth attendants (doctors and nurses) that offer obstetrics and newborn care to a diverse group of women [20]. It is the first level of healthcare in the community, hence they attend to some women who had unsupervised antenatal care but present in labour, those who delivered at home or were managed by traditional birth attendants in unorthodox centres [21-23]. Often when complications arise, some of these cases present to health centres for immediate obstetrics and or newborn care [24]. These emergency situations regularly require the experience of primary health care workers to skillfully manage obstetric complications and perform essential newborn care practices including neonatal resuscitation before further referral to secondary/ tertiary facilities. They should be prepared at all times by intervening promptly and appropriately to help babies who do not breathe when such cases occur. Anticipation and adequate preparation is an important step in decreasing newborn deaths at the community level [25]. This study therefore set out to assess primary health care workers´ experience of neonatal resuscitation and audit primary health centres for availability of neonatal resuscitation equipment.

## Methods

**Study area:** Rivers State is located in the southern part of Nigeria and comprises 23 local government areas (LGAs) and Port Harcourt Metropolis is the state capital. The projected population of Rivers State for 2015 was 6,592,072 with a large proportion of this total population residing in the capital city [21]. “Port Harcourt Metropolis” consists of Port Harcourt city PHALGA and Obio-Akpor LGAs. Port Harcourt is a cosmopolitan city and has some major Government parastatals, multinational companies, industries, an international airport, local airport and two sea ports. The urban nature of the area together with oil exploration and production activity has caused the influx of people of diverse ethnicities [21]. There are 28 Primary Health Care Centres in Port Harcourt (13 in Port Harcourt city LGA and 15 in Obio-Akpor LGA) that are utilized for obstetric and newborn care services. The facilities offer 24 hours services, 7 days a week.

**Study design:** this was a cross-sectional descriptive study.

**Study population:** Health Care Workers (HCWs) including doctors, nurses and nurse/midwives that offer obstetrics and newborn services to mothers attending PHC Centres in Port Harcourt.

**Inclusion and exclusion criteria**

**Inclusion criteria for health care workers:** health care workers that work in PHC Centres in Port Harcourt, who provide obstetrics and newborn services, and health care workers that gave consent.

**Exclusion criterion for health care workers:** community health extension workers and public health officers.

**Inclusion criterion for primary health centre:** PHC Centres which offer basic obstetrics and newborn care services in Port Harcourt.

**Exclusion criterion for primary health centre:** PHC Centres that offer basic obstetrics and newborn care services in Port Harcourt but did not give consent, are not fully functional, dilapidated or led by Community Health Workers.

**Sample size:** the minimum sample size for this study was determined using the formula for quantitative variables for proportions [26].

$$n=\frac{Z^{2}pq}{d^{2}}$$

Where n = sample size, Z = 1.96 (i.e. 95% confidence interval), d = 0.06 (acceptable margin of error), p = 50% (Assumed proportion of primary HCWs with optimal level of experience in neonatal resuscitation), q = 1-p = (Assumed proportion of primary HCWs with no experience in neonatal resuscitation).

Therefore, n = (1.96^2^* 0.5 * 0.5)/(0.06)^2^ =267. The total number of available HCWs in the PHC Centres that offer obstetric and newborn care services was 168. Hence using Finite Population Correction (FPC) formula: [26] where n_x_ = Finite population corrected sample size; m = estimated sample size, N = known finite population size.

Therefore, $\eta x=\frac{\frac{m}{1+(m-1)}}{N}$. nx =(267/(1+(267-1)))/168 = 103. Hence a minimum of 103 HCWs was required for this study.

### Sampling technique

**Selection of PHC centres:** all 28 PHC Centres in Port Harcourt Metropolis met inclusion criteria and were included.

**Selection of health care workers:** stratified sampling by proportionate allocation was used to randomly select primary health care workers. Both local government areas had a total of 168 HCWs. In each PHC Centre, the workers were stratified by cadre into doctors and nurse/midwives. Each centre had two to three doctors and between two to twelve Nurse/midwives. Proportionate allocation was used to achieve the number of HCWs to be selected in each health facility and then each HCW was selected by simple random sampling by balloting. At each facility to minimise bias, the names of HCWs that met inclusion criteria were obtained from the scheduled duty roster and numbers assigned to each name. Then the required number needed to comprise the sample group as calculated by proportionate allocation formula was attained by picking at random after balloting.

**Field researchers:** the researchers are trained, certified neonatal resuscitation programme train-the-trainer’s facilitators of the paediatric association of Nigeria and have been involved in trainings at both state and national levels.

**Tool for obtaining socio-demographic characteristics of health care workers:** semi-structured, self-administered questionnaire was used to collect data about all recruited participants. It included information on the socio-demographic characteristics of the health worker, years of practice (since qualification), the average number of births per month and previous use of bag and mask, suction devices, stethoscope in resuscitation in the preceding six months and whether a neonatal resuscitation flow chart was available in the practicing facility.

**Assessment of primary health centres for availability of neonatal resuscitation equipment:** a data collection Proforma adapted from the standard NRP textbook was used to take an audit of the neonatal resuscitation equipment identified at each PHC centre. The equipment was grouped into categories A and B and were assessed for availability and functionality. Category A assessed the availability of essential items including: mucus extractor or suction apparatus, Infant bag and mask, towels or cloth for newborn, newborn resuscitation table. Category B assessed Availability of priority items: Syringes (2 ml, 5 ml, and 10 ml), stethoscope for use in newborns, source of warmth, resuscitation flow chart and oxygen source.

**Study procedure and data collection:** the lead researcher visited the 28 primary health centres in April 2018 and held a meeting with Key HCWs. The meeting was to inform the Facility Heads about the research. Letters of permission from the Rivers State Primary Health Care Management Board (RSPHCMB) and University of Port Harcourt Teaching Hospital (UPTH) Ethical committee were shown. After which health care workers were recruited. Formal consent was obtained from all selected participants and questionnaires were administered. Audit of neonatal resuscitation equipment in the PHC centres in Port Harcourt and Obio-Akpor LGAs was conducted in April 2018 using a proforma adapted from the American Academy of Paediatrics NRP manual on neonatal resuscitation 6th edition. The researchers filled the proforma at each health facility visited and checked every equipment for both availability and functionality.

**Data analysis:** data from the study were analyzed using the Statistical Package for Social Sciences (IBM SPSS Statistics), version 22.0 with level of significance fixed at a p value of < 0.05. The demographic characteristics of the health workers are displayed on tables. Continuous variables were expressed as means and standard deviations and categorical variables expressed as frequency tables, proportions and charts.

**Data management and storage:** all health care workers enrolled in this study were given participants´ study numbers. The access to data was strictly in the custody of the researchers.

**Ethical consideration:** ethical approval for the study was obtained from the University of Port Harcourt teaching hospital ethics committee and the Permanent Secretary of the Rivers State Primary Health Care Management Board. Consent was obtained from each health care worker enrolled and were allowed to withdraw at any time. All information, including personal details were handled with confidentiality.

## Results

All one hundred and six health care workers who enrolled in this study completed the questionnaires, giving a completion rate of 100% and all 28 PHC centres were visited and availability and functionality of neonatal resuscitation equipment assessed.

**Sociodemographic characteristics of health care workers:** the twenty-two doctors that enrolled in this study were medical officers. Of the 84 (79.2%) nurses enrolled, 50 (59.5%) were nursing officers I/II, 19(22.6%) were senior and principal nursing officers and 15(17.9%) were assistants/ chief nursing officers. One hundred and three (97.2%) of the health care workers recruited were females. The mean age of the health care workers was 38.67 ± 8.14years. The difference in mean ages between males and females was not statistically significant (t = 0.431, p = 0.667). Table 1 shows the sociodemographic characteristics of the health care workers.

**Table 1:** socio-demographic characteristics of the health care workers

Variables	Frequency	Percentage
**Age category**		
≤30 years	17	16.0
31-40 years	57	53.8
41-50 years	20	18.9
>50 years	12	11.3
**Profession**		
Doctors	22	20.8
Nurses	84	79.2

**Distribution of years of practice among health care workers:** Table 2 shows the distribution of years of practice among the health care workers. The mean years of practice among health care workers was 11.72 ± 9.22 years and ranged between one and 35 years. Health care workers with 6-10 years´ experience constituted the highest proportion of the study group.

**Table 2:** distribution of years of practice among health care workers

Years of job practice	Frequency	Percentage
1-5 years	28	26.4
6-10 years	41	38.7
11-15 years	14	13.2
>15 years	23	21.7
**Total**	**106**	**100.0**

**Distribution of health care workers´ experience in neonatal resuscitation:** Table 3 shows the distribution of health care workers´ experience in neonatal resuscitation training. Three-eighths 40 (37.7%) of the health care workers had a previous training in newborn resuscitation. Half 20 (50%) of these, had a neonatal resuscitation training within the previous three years. Table 4 shows average births per month and health care workers´ experience with use of basic neonatal resuscitation equipment during resuscitation. The average number of babies delivered by the health care workers varied widely. Nearly half, 49 (46.2%) of them attend to an average of 11-20 deliveries per month. Although all health care workers had been involved in resuscitating newborns, only half use stethoscopes for evaluation during resuscitation. Table 5 shows health care workers´ experience with use of bag and mask. The table highlights that six months prior to the study, 65 (61.3%) of health care workers did not use a bag and mask (resuscitator device) during resuscitation and 22 (20.8%) had done so, on only one to four babies.

**Table 3:** health care workers experience on neonatal resuscitation training

Variables	Frequency	Percentage
**Ever received training on new born resuscitation(n=106)**		
Yes	40	37.7
No	66	62.3
**How long ago was last training received (n = 40)**		
1-3 years	20	50.0
4-6 years	12	30.0
>6 years	8	20.0

**Table 4:** health care workers’ experience with basic newborn resuscitation equipment

Variables	Frequency	Percentage
**Average number of births attended to in a month**		
1-10	6	5.7
11-20	49	46.2
21-30	32	30.2
31-40	10	9.4
>40	9	8.5
**Ever resuscitated newborns**		
Yes	106	100.0
No	0	0.0
**Ever used stethoscope during new born resuscitation**		
Yes	53	50.0
No	53	50.0
**Ever used bulb syringe during new born resuscitation**		
Yes	90	84.9
No	16	15.1
**Have protocol (flow chart) for new born resuscitation in practice centre**		
Yes	19	17.9
No	87	82.1

**Table 5:** health care workers’ experience with use of bag and mask

Variables	Frequency	Percentage
**Ever Used of bag and mask (resuscitator) during new born resuscitation**		
Yes	58	54.7
No	48	45.3
**Number of babies resuscitated using bag and mask in the past 6 months**		
None	65	61.3
1-4	22	20.8
5-8	13	12.3
9-12	1	0.9
>12	5	4.7

**Figure 1:**
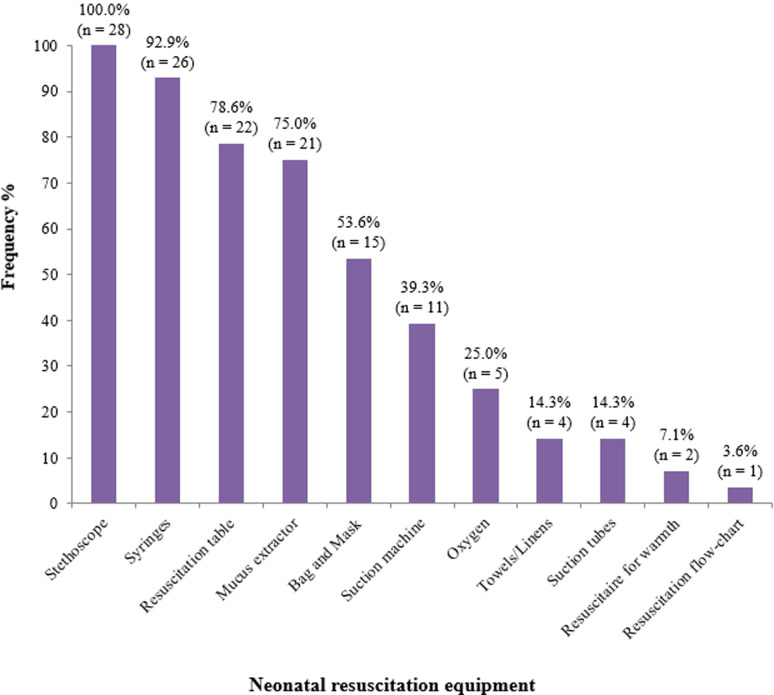
basic neonatal resuscitation equipment available in the PHC centres

**Availability of neonatal resuscitation equipment in PHC centres:** twenty-eight PHC Centres were assessed for resources for neonatal resuscitation. All the health facilities provided some obstetric care including ANC, vaginal deliveries, Family planning but none performed caesarean sections. All PHC centres provided some form of children out-patient clinics and newborn care. Figure 1 is a bar chart showing the neonatal resuscitation equipment available in the PHC centres. Only 15 (53.6%) of the health centres had newborn-specific bag and masks that were functional, 11 (39.3%) had suction machines and 5 (25%) had oxygen cylinders but none had oxygen readily available for use.

## Discussion

This study has shown that the overall experience of newborn resuscitation among primary health care workers in Port Harcourt is very limited. Our findings suggest the possibility that babies born at PHC Centres are inappropriately evaluated according to the NRP or HBB guidelines. The limited use of suction devices, stethoscopes to access heart rates, oxygen or bag and mask resuscitation device during resuscitation, absence of HBB or NRP algorithms at delivery rooms buttress our assertions and is worrisome. Even the relatively low proportion of health care workers that had used bag and mask resuscitation devices had done so in very few births. While the finding may suggest babies born at the PHC centres may be born without complications, it may in fact highlight the inexperience of primary health care workers in neonatal resuscitation. The Rivers State Government had refurbished some of the health centres, but may not have taken into cognizance the possibility of primary health care workers´ inability to effectively use some of the equipment for newborn resuscitation. Our findings therefore bring to fore the need for trainings and refresher training on basic newborn resuscitation among these cohort of health care workers. This study also revealed shortage of the essential commodities needed for basic neonatal resuscitation in PHC Centres in Port Harcourt metropolis. Although some health care facilities have resuscitation devices, it was observed that some health workers had no knowledge about the device or how it functions. A few health workers realized its availability only after the impromptu audit for neonatal resuscitation equipment in their centres. Majority had make-shift resuscitation beds and although stethoscopes were available, were not paediatric-specific. The findings suggest that some PHC Centres are not adequately equipped to provide basic neonatal resuscitation, considering the importance of ventilation in averting the negative effects of birth asphyxia. Our finding agrees with reports of unavailability of neonatal resuscitation equipment in other health care facilities in Nigeria [6,27-29] and other primary health centres in other resource poor settings [17, 30, 31]. The facts that some health workers were unable to identify neonate-specific bag and masks and some of the resuscitator devices were locked up in stores instead of being available at each newborn´s birth suggests underutilization of material resources already made available by the Government of Rivers State, lack of accountability of newborn resources, lack of preservice or in-service trainings, or improper stock taking/ handover with successive administrations at the PHC Centres. This study therefore has identified some gaps; the limited experience of neonatal resuscitation among primary health care workers in Port Harcourt and the fact that re-stocking of neonatal equipment alone will not be sufficient. It suggests that both supplies of equipment for newborn resuscitation and training/re-training of health care workers at the community level be emphasized.

**Limitation:** this study did not assess the skills of the health workers with live babies or simulations, however, it is clear that even where the knowledge and skills for initiating positive pressure ventilation is available, lack of appropriate equipment may hinder progress and successful outcome during resuscitation.

## Conclusion

The very limited experience of primary health care workers in basic neonatal resuscitation and the shortage of basic neonatal resuscitation equipment at the PHC Centres provide a possible explanation for the high neonatal mortality rates in Rivers State and Country. We therefore recommend that nurses/midwives and doctors in PHC Centres should be trained in basic neonatal resuscitation. This is particularly imperative to circumvent the need for referrals to secondary/ tertiary facilities and prevent the economic and physical burden in the management of neonatal encephalopathy and its long term sequelae. The Rivers State Government and all stakeholders should ensure appropriate neonatal resuscitation equipment are in the State´s procurement and distribution lists and are supplied to the PHC Centres. Health care workers at PHC Centres should ensure that neonatal resuscitation equipment are kept in good working condition and made available at all births. With the commendable efforts made by the Rivers State Government to achieve universal health coverage and ensure sustainable quality health by refurbishing PHC Centres, training of primary health care workers should also be strengthened commensurately.

### What is known about this topic

- Newborn deaths from birth asphyxia remain high in resource limited settings like Nigeria;
- The presence of birth attendants skilled in newborn resuscitation can prevent asphyxia related complications;
- In Nigeria, deliveries occur more at PHC Centres and homes but the presence of birth attendants skilled in newborn resuscitation in lacking.

### What this study adds

- Confirms a limited experience of basic neonatal resuscitation among Primary Health Care Workers in Rivers State, Nigeria and no other study has been previously published;
- Highlights the urgent need for training of health workers in neonatal resuscitation;
- Stocking Primary Health Centres with newborn resuscitation equipment alone is insufficient.
